# Cobimetinib Plus Gemcitabine: An Active Combination in KRAS G12R-Mutated Pancreatic Ductal Adenocarcinoma Patients in Previously Treated and Failed Multiple Chemotherapies

**DOI:** 10.1089/pancan.2021.0006

**Published:** 2021-10-13

**Authors:** Bach Ardalan, Jose Azqueta, Danny Sleeman

**Affiliations:** ^1^ Department of Hematology Oncology, Sylvester Comprehensive Cancer Center, Miami, Florida, USA.; ^2^ Department of Surgical Oncology, Sylvester Comprehensive Cancer Center, Miami, Florida, USA.

**Keywords:** pancreatic cancer, KRAS-mutant, KRAS G12R, MEK inhibitor, next-generation sequencing

## Abstract

**Purpose:** The KRAS proto-oncogene is involved in the RAS/MAPK pathway. KRAS is present in the wild type or mutated forms. The oncogene KRAS is frequently mutated in various cancers. At the time that amino acid glycine is mutated, KRAS protein acquires oncogenic properties that result in the tumor cell growth, proliferation, and cancer progression. There has been limited understanding of the different mutations at codon 12. The consequences of such mutations is not fully understood. Various G12X mutations in pancreatic cancer patients have been examined, with the most common mutations being G12D (40%), G12V (30%), and G12R (15–20%). Now we are understanding that G12X mutations in the KRAS are not all equal.

**Methods:** In a single-arm exploratory study, we accrued 13 KRAS-G12X-mutated pancreatic patients (KRAS G12D, G12V, and G12R). They were divided into two groups: group 1 consisted of seven patients with G12D and G12V and group 2 included six patients with the KRAS G12R mutation. All patients were treated with the combination of gemcitabine at 1250 mg/m^2^ intravenous weekly for 3 weeks and oral cobimetinib 20 mg b.i.d. for 3 weeks. This was followed by a week of rest before the initiation of the next cycle.

**Results:** In the first cohort, seven patients were on treatment, all of whom progressed and died within the 2 months of the study. In the second cohort, one of six patients achieved partial response, and five achieved stable disease. Median progression-free survival was 6 months (9% confidence interval 3.0–9.3 months) and overall survival has been reached at 8 months. Common adverse reactions included rash, fatigue, nausea, and vomiting (grades 2 and 3). Cancer antigen CA19-9 decreased by >50% in all group 2 patients.

**Conclusion:** Our pancreatic cancer patients were heavily pretreated (all had received FOLFIRINOX and gemcitabine/nab-paclitaxel) before the entry into our trial. Upon entry into our trial, all patients were treated with the combination of gemcitabine and oral cobimetinib. Therefore, this constituted the second exposure of the patients to gemcitabine. This study illustrates a new discovery, which can potentially target 15–20% of pancreatic cancer patients and allow for a significant improvement in their prognosis. We will be conducting randomized phase II trials to substantiate our findings.

## Highlights

- Not all KRAS mutations are equal
- G12R patients may have favorable outcome to the combination of MEK inhibitors and chemotherapy

## Introduction

Pancreatic adenocarcinoma is a leading cause of cancer death and is responsible for 6% of all cancer deaths.^1^ Life expectancy of pancreatic cancer patients has improved slightly, with a 5-year overall survival (OS) of ∼9%.^2^ Gemcitabine was the first agent Food and Drug Administration approved for this malignancy. In a randomized study, comparing gemcitabine with 5-fluorouracil, gemcitabine monotherapy resulted in the longer survival as compared with 5-fluoruracil. In 2010, a significant improvement in survival was achieved with the FOLFIRINOX trial.^3^ Patients treated with the combination of 5-fluorouracil, oxalipaltin, and irinotecan showed a significant OS compared with the patients on monotherapy with gemcitabine, with a median OS of 11.1 versus 6.8 months. With the development of gemcitabine and nab-paclitaxel, it is now customary for the patients who have failed FOLFIRNOX to be placed on the combination of gemcitabine.^4^ This year, ∼57,770 new pancreatic cancer cases are expected to be diagnosed. The majority of these patients 95% will demonstrate KRAS oncogene mutation.^5–7^ We have submitted the tissue samples for the next-generation sequencing using the Illumina DRAGEN platform, to confirm the mutational subset of each patients before the beginning of the therapy.^8^ There are five common mutations of KRAS G12X: these are G12D, G12V, G12C, G12S, and G12R.^9^ Moreover, in pre-clinical studies of patient-derived xenograft-derived tumors transplanted in mice and treated with MEK inhibitors plus and minus chemotherapy, there was a greater degree of tumor regression with the combined therapy.^10^

## Methodology

Patients were eligible if they met the following criteria: pathological proven adenocarcinoma of pancreas, previously have received and failed the two standard pancreatic cancer treatments (namely FOLFIRINOX and gemcitabine/nab-paclitaxel), Easton Cooperative Oncology Group score of 0–1, absolute neutrophil count >1500 mm^3^, hemoglobulin level >9 g/dL, platelets count of >100,000 mm^3^, total bilirubin level <2 mg/dL, and creatinine level <1.5 mg/dL. Patients enrolled all had next generation sequencing-confirmed KRAS G12X mutations; patients who did not have a mutation on codon 12 (i.e., KRAS G13) and KRAS wild-type (WT) were excluded from the study. Those with serious mental disorders were ineligible. All the patients enrolled in the study were informed and signed the consent forms. All procedures performed in this study involving a human participant adhered to the 1964 Declaration of Helsinki and its later amendments. Collection of information in this report complied with the Health Insurance Portability and Accountability Act of 1996. All patients received 2 g of dexamethasone intravenous as antiemetic in addition to zofran before the treatment. Gemcitabine 1250 mg/m^2^ was administered over 30 min on days 1, 8, and 15. The treatment was continued in repeated 28-day cycles as long as the treatment was tolerated until the progression of the disease. Concurrently, cobimetinib at the dose of 20 mg b.i.d. was given orally for 3 weeks and 1 week off before the start of the next therapy. Granulocyte colony stimulation was used as it deemed necessary. Dose reductions or treatment delays were performed as described in Table 1.^4^ The treatment was delayed when absolute neutrophil count was <1500 mm^3^, hemoglobin level <9.0 g/dL, platelet count of <100,000 mm^3^, total bilirubin >3 mg/dL, aspartate transaminase greater than threefolds normal, and creatinine levels >1.5 mg/dL. Other side effects requiring dose reductions were grade 3 mucositis or diarrhea.

**Table 1.** Dose Modification

Dose level	Gemcitabine, mg/m^2^	Cobimetinib, mg
Full dose	1250	20 mg b.i.d. orally
Level 1	1000	20 mg once per day orally
Level 2	750	None

## Assessment

Throughout the entire treatment course, patients were assessed for the overall general conditions and any possible side effects, by physical and blood examinations that included complete blood counts and blood chemistry. The examinations were performed once a week by the attending physicians. The treatment response was assessed by a radiologist by comparing computed tomography scan and positron emission tomography scans every 8 weeks. Adverse events were scored using the National Cancer Institute Common Terminology Criteria for adverse version 4.0. The radiological tumor response was evaluated using the Response Evaluation Criteria in Solid Tumors version 1.1. The response rate was defined as the best observed response rates. OS was calculated from the date the patients were removed from the second line chemotherapy and placed on gemcitabine and cobimetinib.

## Results

Between November 2018 and January 2020, 13 patients were placed on this program. The basic patient characteristics are given in Table 2. This a balanced study with respect to the age, gender, performance status, and the extent of the disease. Both groups had received similar prior chemotherapy with FOLFIRINOX and gemcitabine/nab-paclitaxel. Moreover, the response of the two groups to FOLFIRINOX and gemcitabine/nab-paclitaxel were similar. In this small study, the response to gemcitabine/cobimetinib in the two arms was different, namely in group 2 (G12R), there was one partial response and five stable disease (SD), whereas in group 1 (G12D, G12V), there were seven progression of disease.

**Table 2.** Patient Characteristics

Characteristics	Group 1 (G12D and G12V)	Group 2 (G12R)
Age (median range)	64 (55–71)	61 (49–72)
Gender (male/female)	4/3	3/3
ECOG (0/1)	0/1	0/1
CA19-9 (median range)	1977	2300
Disease extent	Advanced	Advanced
Site of metastases	Lung/liver	Lung/liver
Number of FOLFIRINOX	4–6	4–6
Number of gemcitabine/nab-paclitaxel	2–4	2–4
Response to FOLFIRINOX	CR: 0	CR: 0
	PR: 1	PR: 1
	SD: 4	SD: 4
	PD: 2	PD: 1
Response to gemcitabine/nab-paclitaxel	CR: 0	CR: 0
	PR: 1	PR: 1
	SD: 1	SD: 2
	PD: 5	PD: 3
Response to gemcitabine/cobimetinib	CR: 0	CR: 0
	PR: 0	PR: 1
	SD: 0	SD: 5
	PD: 7	PD: 0
Median survivorship from the start of the study in months	2	8

Toxicity profile is given in Table. Hematological and nonhematological toxicities are listed and equivalent in the two arms of the study. Moreover, grade 3 toxicities were minor and seen in both arms of the study. Notable nonhematological toxicities were nausea/vomiting, mucositis, fatigue, and diarrhea.

CR, complete response; PD, progressive disease; PR, partial response; SD, stable disease.

## Discussion

This is an exploratory study evaluating the combination of a MEK 1 and 2 inhibitor with chemotherapy in patients with advanced pancreatic cancers who have received and failed the two standard chemotherapy regiments. This is the *first* report of the use of second-generation MEK 1 and 2 inhibitor in combination with a chemotherapy agent that has generated a limited increased survival in a small subset of patients with pancreatic cancer with a known KRAS mutation. Ninety-five percent of patients with pancreatic cancer demonstrate KRAS molecule mutations. It is only recently, through tumor genomic analyses, it is known that most genetic mutations in the KRAS occur in codon 12.^5–7^ Mutations in the other codons are uncommon.^7^ In codon 12, there are five common mutations and these mutations constitute >90% of KRAS mutations. The most important mutations in codon 12 are G12C/V/D/S/R. G12D is the most common mutation (51%), followed by G12V (30%), G12R (15%), and G12C/S (2% each). Less frequent mutations, constituting <1%, are observed at codon 13 (i.e., G13C/D/P/S) and codon 61 (Q61H/K/R). For the past decade, several attempts have been made to target RAS pathway, however, most attempts have failed.^11–13^ Many agents have shown inability to bind to the small binding pockets of KRAS, and high guanine triphosphate concentration has rendered KRAS protein undruggable. Furthermore, the canonical signaling of downstream targets of KRAS-RAF, MEK, and subsequently ERK-creating BYPASS pathways for RAS targeting.

Recently, the novel compound sotorasib has demonstrated activity, as it occupies the His95 groove near the cysteine pocket to maintain high levels of inactive KRAS.^14^ Initial results are encouraging in lung cancers that harbor KRAS G12C mutation. In pancreatic adenocarcinoma, KRAS G12C mutation is <2%. KRAS G12R is most commonly seen in pancreatic tumors versus other tumors of humans (15%). The phosphoinositide 3-kinases (PI3k)/phosphatase and tensin homolog (PTEN)/protein kinase B (Akt)/mammalian/mechanistic target of rapamycin complex 1 (mTORC1) is another key pathway activated in pancreatic adenocarcinomas and the latter pathway is associated with KRAS. Monotherapy targeting of P13k, AKT, and mTOR has not been successful in RAS-mutated pancreatic cancers. Dual PI3K in combination with RAF-MEK-ERK inhibitors is currently being tested. A randomized phase II study of selumetinib, a MEK inhibitor, and MK-2206, an AKT inhibitor, failed to show any benefit compared with modified FOLFOX therapy in patients who had failed gemcitabine-based therapy.^15,16^

In the past, several attempts have been made to incorporate various MEK inhibitors into clinical practice. In one randomized double-blinded placebo-controlled trial of trametinib in combination with gemcitabine in patients untreated with pancreatic cancer, the results were negative (OS, 8.4 months for the combination versus 6.7 months for gemcitabine plus placebo). However, there are several pitfalls in this study. First, >20% of patients in either arms of the study were KRAS WT. The WT should not constitute >5%. Furthermore, no mention has been made whether any of the KRAS WT patients were also BRAF V600. Second, the number of KRAS-mutated patients examined were in 50 patient range that would translate into seven patients with KRAS G12R in each arm. The mentioned number is based on the frequency of G12R patients in the KRAS-mutated population.^17^

A recent article addresses a study of selumetinib in heavily treated pancreatic cancer patients as a single agent. All patients were KRAS G12R. After a median follow-up period of 8.5 months, three patients had SD for >6 months. Median progression-free survival was 3 months and median OS was 9 months.^18^

**FIG. 1.**
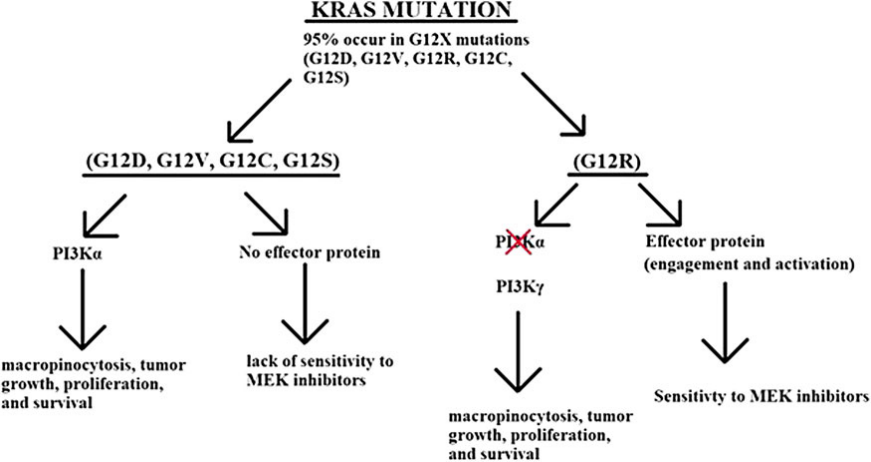
KRAS pathways. All the common mutations G12D, G12V, G12C, and G12S operate through PI3K alpha, and have no effector protein to interact with the MEK inhibitors. In contrast, G12R operates through PI3K gamma and HAS an effector protein that interacts with MEK inhibitor. This interaction is augmented in the presence of gemcitabine.

In this study that should be considered an exploratory study, we have enrolled patients who have failed the two standard chemotherapies for pancreatic cancer. All patients had genomic sequencing on their tumor or liquid biopsy performed, confirming the KRAS mutation before commencing. Subsequently the patients were divided into two groups: group 1, all mutations of codon 12, that is, G12D or G12V but not mutations in the other codons; group 2, only KRAS G12R-mutated patients. Both cohorts used the same dose of cobimetinib, 20 mg b.i.d. Eligible patients were treated as already outlined. As indicated in Table 3, toxicities in both arms were comparable, however, we observed difference in the response and survival in the two groups.^19^ As is shown in Figure 1, we have the following mechanistic explanations for this difference. First, in the non-G12R, PI3k alpha is active that leads to micropinocytosis, tumor growth, and proliferation. In G12R mutations, PI3K alpha is not active and is replaced by PI3K gamma. Second, in KRAS non-G12R mutations, there is no effector protein engagement or activation that leads to the lack of activity to MEK inhibitors. However, in KRAS G12R mutations, there is an effector protein engagement and activation that leads to sensitivity to MEK inhibitors.^20^ Two contradictory clinical studies have noted different responses from KRAS G12R-mutated patients: one study reported a better OS (G12D = 6 months, G12V = 9 months, G12R = 14 months) and the second study indicated both G12R and G12D patients have an overall poor prognosis.^21,22^ Therefore, it is not entirely clear that G12R mutations have a better prognosis than the other mutations.

**Table 3.** Toxicity Profile

	KRAS (G12D/V)			KRAS (G12R)		
	*n* (%)	Greater and equal Grade 3	%	*n* (%)	Greater and equal Grade 3	%
Hematological toxicities						
Neutropenia	3 (42)	1	14	2 (33)	1	16
Febrile neutropenia	1 (14)	1	14	1 (16)	1	16
Thrombocytopenia	2 (28)	1	14	1 (16)	1	16
Anemia	3 (42)	1	14	2 (33)	1	16
Nonhematological toxicities						
Nausea/vomiting	4 (57)	2	28	3 (50)	1	16
Anorexia	3 (42)	1	14	2 (33)	1	16
Mucositis	4 (57)	2	28	3 (50)	1	16
Fatigue	4 (57)	1	14	2 (33)	1	16
Diarrhea	4 (57)	1	14	2 (33)	1	16

However, we believe that with the addition of gemcitabine to an MEK inhibitor, cell growth and proliferation are favorably reduced. We are looking forward to conduct a formal phase II study to confirm our findings.

## Conclusions

In pancreatic cancer, G12R mutations are more responsive when treated with a combination of MEK inhibitors and chemotherapy.

## Data Availability

All data generated or analyzed during this study are included in this publication.

## Employments

The authors' employment has no invested interest that may gain or lose financially from publication of the article.

## Abbreviations Used

CA19-9: carbohydrate antigen 19-9 is a blood group antigen that serves as a tumor marker in patients with gastrointestinal cancers
CR: complete response
KRAS: oncogene in the MAPK pathway in the Ras family of oncogenes
MEK 1 and MEK 2: MEK is an MAPK/ERK kinase belonging to the regulatory that acts as a tyrosine and serine/threonine kinases
OS: overall survival
PD: progressive disease
PR: partial response
SD: stable disease
WT: wild-type
